# Exploring Novel Applications for Hydrogels Derived from Modified Celluloses

**DOI:** 10.3390/polym16040530

**Published:** 2024-02-16

**Authors:** Feiyang Wang, Aldo Borjas, Aldrin Bonto, Alina Violeta Ursu, Maxime Dupont, Jane Roche, Cédric Delattre

**Affiliations:** 1 Clermont Auvergne INP, CNRS, Institut Pascal, Université Clermont Auvergne, F-63000 Clermont-Ferrand, France; 2 INRAE, Laboratoire de Génétique, Diversité et Écophysiologie des céréales (GDEC), Université Clermont Auvergne, F-63000 Clermont-Ferrand, France; 3 Department of Chemistry, De La Salle University, 2401 Taft, Avenue, Manila 0922, Philippines; 4 Institut Universitaire de France (IUF), 1 Rue Descartes, 75005 Paris, France

**Keywords:** wheat straw, microcellulose, TEMPO oxidation, hydrogel, rheological, printability

## Abstract

The valorization of lignocellulosic biomass by-products holds significant economic and ecological potential, considering their global overproduction. This paper introduces the fabrication of a novel wheat-straw-based hydrogel and a new microcellulose-based hydrogel through 2,2,6,6-tetramethylpiperidinyl-1-oxy (TEMPO) oxidation. In this study, Fourier transform infrared (FTIR) analysis was employed for the detection of carboxyl groups, neutralization titration was conducted using a conductivity meter, viscosity analysis was performed using a rheometer, and transmittance analysis was carried out using a spectrophotometer. Two novel hydrogels based on TEMPO oxidation have been developed. Among them, the bio-based hydrogel derived from oxidized wheat straw exhibited exceptional printability and injectability. We found that the oxidation degree of microcellulose reached 56–69%, and the oxidation degree of wheat straw reached 56–63%. The cross-linking of 4% oxidized wheat straw and calcium chloride was completed in 400 seconds, and the viscosity exceeded 100,000 Pa·s. In summary, we have successfully created low-cost hydrogels through the modification of wheat straw and microcellulose, transforming lignocellulosic biomass by-products into a sustainable source of polymers. This paper verifies the future applicability of biomass materials in 3D printing.

## 1. Introduction

The rapid development of the social economy has improved the quality of human life, but it has also led to environmental challenges. Approximately 21% of the world’s food depends on the wheat crop, and global production needs to be increased to meet the growing demand for human consumption [1]. Wheat straw, as a by-product of food crops, possesses a high cellulose content (around 50%), and its resource utilization can contribute to alleviating environmental issues caused by burning. Lignocellulose is biocompatible, degradable, and readily available, and can be utilized as a biocomposite material in various applications such as food packaging, tissue engineering, and energy regeneration [2,3]. Nevertheless, challenges exist in the reuse of agricultural by-products, such as the degradation of the physical properties of wheat straw when incorporated into polylactic acid-based biocomposites for sustainable food packaging [4]. Hence, wheat straw, as a green biocomposite material, faces challenges related to interfacial adhesion and dispersion properties. Addressing these challenges can involve chemical modification, such as adding hydrophilic groups, or physical modification, like mechanical crushing, to enhance its suitability as a composite packaging material [5]. Numerous methods exist for pretreating biomass materials to enhance their application performance. These approaches include the use of various compatibilizers such as siliconization, alkali treatment, acylation, isocyanate treatment, etc., all of which aim to increase the adhesion and strength of biomass composites [6].

The TEMPO-NaBr-NaOCl system in water at pH 10 quantitatively produces water-soluble sodium polyglucuronic acid when it reacts with water-insoluble polysaccharides such as starch, curdlan, chitin, paramylon, and regenerated cellulose samples under suitable conditions [7]. TEMPO-oxidized polyglucuronic acid prepared from regenerated cellulose and glycans is biodegradable. All artificial polyglucuronic acids prepared through TEMPO catalytic oxidation undergo enzymatic degradation and metabolization into CO_2_ under the influence of microorganisms in natural environments [8]. The carboxyl-containing cellulose prepared by TEMPO oxidation is water-soluble, and the repulsion between the negative charges of the carboxyl groups can create a stable suspension. This property makes it suitable for applications in various fields such as food, medicine, papermaking, plastics, and energy [9,10].

Hydrogels find extensive application in tissue engineering owing to their distinctive environmental responsiveness, flexibility, and biocompatibility [11]. For instance, certain hydrogels play a transformative role in tissue engineering by serving as biomimetic scaffolds that replicate the mechanical characteristics of native tissues, thereby enhancing integration with surrounding tissues and improving cell viability. A diverse array of hydrogels with tailored properties has been developed to address the specific needs of tissue engineering applications for cartilage, bone, and blood vessels [12]. Polysaccharides are regarded as ideal materials for hydrogels owing to their natural degradability, cost-effectiveness, and excellent biocompatibility [13]. They can be obtained through the fermentation and purification of various sources, including animals, plants, and microorganisms (such as polysaccharides, peptides, and proteins), making them suitable for industrial production [14]. A substantial body of research suggests that the 2,2,6,6-tetramethyl-1-piperidine epoxy (TEMPO) radical can effectively catalyze the primary hydroxyl group in polysaccharides [15]. This catalytic capability has found successful applications in various cellulose-based products [16].

Cellulose stands out as the predominant biopolymer, characterized by highly stereoregular, hydrogen-bonded beta-(1,4)-glycoside-linked D-glucose chains [17]. This biopolymer has been extensively explored for green applications owing to its biocompatibility, sustainability, and economic attributes [18]. The abundance of easily modifiable hydroxyl groups in cellulose makes it amenable to oxidation reactions, resulting in the formation of carboxylate groups [19]. This transformation imparts new functionalities, such as cross-linking with divalent metals to create hydrogels. Consequently, cellulose is regarded as a promising and versatile modifiable polysaccharide in the field of tissue engineering [20]. Cellulose can be obtained from plants such as cotton, kenaf, ginger, or straw [21]. However, the separation process often involves the use of harsh chemicals, leading to environmental pollution. Therefore, while the rapid development of the cellulose industry has provided affordable and durable products for human use, it has also introduced environmental challenges. We aimed to explore renewable, natural polymers as alternatives to mitigate pollution [22].

Agricultural biomass residues such as wheat straw and rice straw are often discarded or burned due to their perceived low intrinsic value, contributing to environmental pollution. However, in reality, these wastes can serve as an abundant and inexpensive source of renewable lignocellulosic biomass. In recent years, various applications of wheat straw as an agricultural by-product have been explored, including pulp production [23], food packaging [4], and nanocellulose products [24]. Among them, the main way is to extract cellulose from wheat straw. The commercial industry uses raw materials including bagasse and wheat straw. Unbleached pulp and other agricultural residues for the preparation of lignocellulosic nanofibers. The conversion techniques include physical (ultrasound, refining, microfluidization) and chemical methods (acid hydrolysis, enzymatic hydrolysis, pulping, and bleaching reactions) or a combination [25,26]. Nevertheless, existing methods are cumbersome, reliant on specialized equipment, and often lack environmental friendliness. Common pretreatment methods include enzymatic hydrolysis, high-intensity ultrasonication, mechanical treatment, and various other techniques [27]. In our experiment, we aimed to streamline the process by exploring the direct use of raw materials for oxidation without any preliminary treatment. The objective was to create a novel type of hydrogel for future 3D printing applications, simplifying the overall procedure, and potentially reducing the environmental impact associated with traditional approaches.

The valorization of lignocellulosic biomass wastes holds significant economic and ecological potential, given their global overproduction. Recycling these wastes enables circular production strategies in agriculture and forestry [28]. Wheat straw, primarily composed of cellulose, lignin, and hemicellulose, is particularly noteworthy. Its abundant hydroxyl functional groups make it valuable for applications such as bioadsorption and hydrogel production [29]. Cellulose hydrogels stand out as a superior material due to their high water absorption efficiency, charged groups, and porous structure. While the preparation of cellulose hydrogels offers an ideal strategy for utilizing straw, it necessitates pretreatment to remove a significant portion of the lignin and hemicellulose present [30]. TEMPO-mediated oxidation of wood pulp, coupled with slight mechanical disintegration, results in the production of cellulose nanofibers during the oxidation process [31].

The primary goal of this study was to create natural hydrogels using microcellulose and wheat straw and assess the impact of TEMPO-mediated oxidation on the physicochemical properties of these hydrogels. The optimal chemical composition of the hydrogels was determined by considering factors such as viscosity, crosslinking time, injectability, light transmittance, and printable properties. This comprehensive approach aims to tailor the hydrogels for various applications in tissue engineering from abundant lignocellulose plant biomasses/by-products.

## 2. Materials and Methods

### 2.1. Materials

In this experiment, the microcellulose was extracted from cotton from Sigma-Aldrich (St. Quentin Fallavier Cedex, France) and the particle diameter was about 20 microns. Wheat straw was sourced from GDec Lab in 2023, with a composition including 24.5% hemicellulose, 38.8% cellulose, and 11.3% lignin.

### 2.2. TEMPO Oxidation

Microcellulose (6 g) was dissolved in 90 mL of water, the pH was adjusted to 10 with NaOH (1 M), and then the volume of the solution was made up to 100 mL with water, and the reaction temperature was kept at room temperature and homogenized for 1 h. TEMPO (51.96 mg), NaBr (1.152 g), and NaOCl (60 mL at 9.6%) were added to start oxidation. When TEMPO and NaBr are completely dissolved, start adding NaOCl. The timing starts with the addition of NaOCl and the reaction time is 3–24 h. NaOCl is added slowly while using NaOH (10 M) to keep the pH of the solution at 10–11. The reaction was quenched by adding ethanol (99%, 60 mL) after 3–24 h and neutralized with HCl (5 M) to pH around 7. Transfer the solution to a centrifuge tube, after centrifugation (10,000× *g*, 10 min, 4 °C), remove the supernatant, wash the pellet with 50 mL of MilliQ water, then centrifuge at 10,000× *g*, 4 °C for 10 min, repeat 5 times. The pellet was then vortexed with 50 mL of ethanol (99%) for 15 min, then centrifuged at 10,000× *g* and 4 °C for 10 min. The washed precipitate was put into the oven, dried at 50 °C for 24 h, and then ground into powder and passed through a 125 μm sieve for subsequent experiments [32].

### 2.3. FTIR Analysis

The raw powdered and oxidized cellulose and wheat straw samples were analyzed in a Fourier transform infrared spectrophotometer (Thermo Scientific™ Nicolet™ iS™5) coupled to ATR Accessory (Thermo Scientific™ iD7) with a monolithic diamond crystal. The spectra were acquired in the 4000–500 cm^−1^ range, with 4 cm^−1^ resolution, 32 scans, and were subjected to background subtraction (atmosphere spectra) [33].

### 2.4. Conductivity

The 2% oxidized microcellulose and wheat straw were dispersed in water for 24 h and sonicated for ten minutes until the full suspension solution was ready. Add 0.5 mL of 0.1 M HCl to 25 mL of a 0.2 wt% suspension to protonate all carboxyl substrates in oxidized cellulose and wheat straw. Subsequently, titrate the suspension with 0.01 M NaOH. Record conductivity values using the Sension+ EC7. The surface charge is then calculated in millimoles per gram (mmol·g^−1^).

(1) σ=cv÷m=c(v1−v2)÷m

where *c* is the NaOH concentration (in M), *m* is the oxi-cellulose and oxi-wheat straw mass in the suspension (in g), and *v*_1_ and *v*_2_ are NaOH volumes (in mL) used from neutralizing the added HCl and carboxylic acid on the oxi-cellulose or oxi-wheat straw, respectively. Oxidation degree calculation:

(2) $$\begin{array}{l} DS=162\sigma ÷(1-80\sigma )\times 5 \end{array}$$

In the formula, 162 is the mass of the glucose motif, 80 is the mass of CH-COO-Na, and 5 is the correction factor.

### 2.5. Cross-Linking Procedure

The powder obtained from 2.2 was used and reconstituted in water as a suspension of 2%, 4%, and 6%, vortexed for 5 min, dissolved for 24 h, and then sonicated for 5 min for later use. The 1 M CaCl_2_ was added to the suspension at 1:9 (*v*/*v*) and vortexed for 5 min to complete cross-linking. The cross-linked hydrogels were tested using the inverted vial method. The cross-linking time was analyzed using a rheometer (G′ and G″ curve intersection for gelation point). The transition from liquid to solid was observed by examining the intersection of the storage modulus and the loss modulus through frequency oscillation.

### 2.6. Rheological Analysis

All rheological measurements were conducted using a Rheology TA Instruments AR-G2 rheometer equipped with a cone plate featuring a 40 mm upper cone with an angle of 4° and a smooth 40 mm lower plate. Frequency sweep tests were performed under a 1% strain across a range of 0.1~100 Hz. The material’s stress was determined through a shear stress sweep ranging from 1% to 100%. Thixotropy time was gauged by initially applying shear stress exceeding that of the oxidized polysaccharide system. Subsequently, the applied stress was reduced below the material’s stress, and the shear rate was monitored over time.

Subject 4% m/m oxidized cellulose and wheat straw to 350 rpm for 24 h in water. Subsequently, use an Ultrasonic Processor UP50H (Teltow, Germany) for 5 min at 30 kHz. (The aim is to mix the suspension by ultrasonication and at the same time open its structure, which is also accompanied by the production of some nanofibers, making it easier to react with the crosslinker and also increasing its light transmittance). Ensure that all subsequent solutions are processed following this established procedure.

### 2.7. Injectable and Printable Analysis

Injectable capacity and printability assessments were conducted using 4% oxidized microcellulose and oxidized wheat straw. For injectable capacity, the material was initially added dropwise into a vial using a 19 G (0.1 mm) needle, and its viscosity was demonstrated through the inverted vial method. Subsequently, a new vial containing 1 M CaCl_2_ was prepared, and the material was injected into the CaCl_2_ using a 0.1 mm needle to compare its state in the vial with and without CaCl_2_. To assess printable capability, a straight line was drawn in an empty Petri dish using a 27 G (0.4 mm) needle, and the stability of the material and its ability to maintain its shape were observed. Subsequently, another straight line was drawn with a 27 G (0.4 mm) needle in a new Petri dish containing 1 M CaCl_2_ to compare its printing capabilities with and without CaCl_2_.

### 2.8. Transmittance Analysis

The transmittance was used to express transparency. The transmittance of oxidized polysaccharides in the wavelength range of 200–800 nm was assessed using a spectrophotometer. Initially, 4% oxidized microcellulose and wheat straw were analyzed, followed by a 5-min ultrasonic treatment, after which the tests were repeated.

### 2.9. Granularity Analysis

The particle size analysis was performed by the Mastersizer 3000 (particle size range from 0.01 to 3500 μm, France, Malvern Company). As mentioned in 2.2, before the experiment begins, all samples are ground and passed through a 125 µm sieve, and for sonicated samples, dissolved in water and then sonicated for five minutes (same as above), then thoroughly dried and then ground then passed through 125 mesh sieve. To conduct the experiment, begin by pouring 100% ethanol into a preheated 500 mL beaker. Once the instrument is adequately preheated, add oxidized cellulose and wheat straw particles until the detected signal reaches within the measurable range and stabilizes gradually. Activate the laser to measure particle size. Post-measurement, rinse the system thoroughly with 100% ethanol, repeating this process five times until no particle prompts are detected. This entire procedure should be repeated three times for robust and reliable results.

## 3. Results

### 3.1. FTIR Analysis

**Figure 1:**
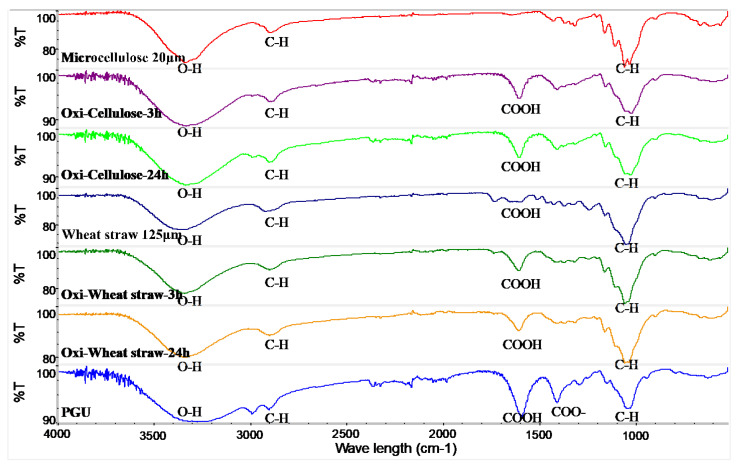
FTIR spectra of oxidized microcellulose, from top to bottom, microcellulose, oxidized for 3 h, oxidized for 24 h, wheat straw, oxi-wheat straw for 3 h, oxi-wheat straw for 24 h, polyglucuronic acid (PGU: bacterial polyglucuronic acid from *S. meliloti* M5N1CS).

To confirm the success of the oxidation reaction, we utilized Fourier transform infrared spectroscopy (FTIR) to analyze the powders post-reaction. The FTIR spectrum of oxidized cellulose exhibits a prominent band at approximately 1610 cm^−1^, indicative of the incorporation of carboxylic acid groups through TEMPO-mediated oxidation (Figure 1). Additionally, the decrease in the OH peak around 3400 cm^−1^ corresponds with the increase in the COO peak, providing further evidence that the TEMPO-mediated oxidation reaction involves the conversion of the hydroxyl group at the C6 position to a carboxyl group. In oxidized wheat straw, it was observed that while some carboxyl groups were initially present in the non-oxidized wheat straw, oxidation further augmented its carboxyl content. Notably, the carboxyl content exhibited an increase corresponding to the extension of the oxidation time.

### 3.2. Conductimetric Titration

**Figure 2:**
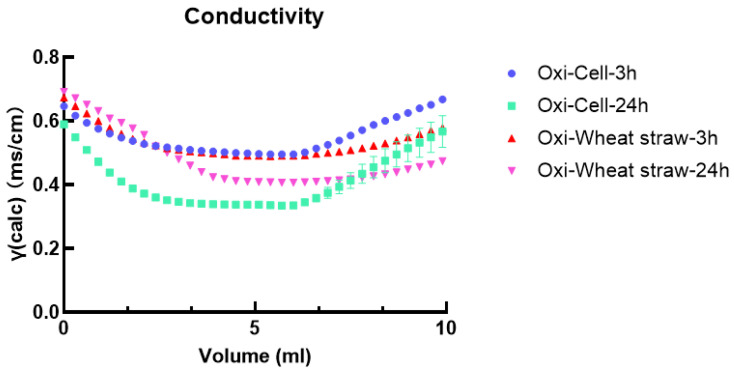
Conductivity analysis of oxidized microcellulose and oxidized wheat straw.

The carboxyl content can be assessed through acid–base titration (Figure 2). In acidic conditions, carboxylic groups react with hydrogen ions, forming carboxylic acids, and under alkaline conditions, carboxylic acids react with hydroxide ions, producing water and a corresponding salt. Through titration of the acid–base solution, the content of oxidized microcellulose and carboxyl groups in wheat straw can be quantified. The findings indicated that the oxidized microfiber had carboxylic acid contents of 56% and 69% for 3 h and 24 h, respectively.

Meanwhile, the oxidized wheat straw exhibited carboxylic acid contents of 56% and 63% for 3 h and 24 h, respectively (Table 1). Hence, the 24-h oxidation will introduce more carboxyl groups to the material, and subsequent comparisons will be conducted under this condition.

### 3.3. Gelation Analysis

**Figure 3:**
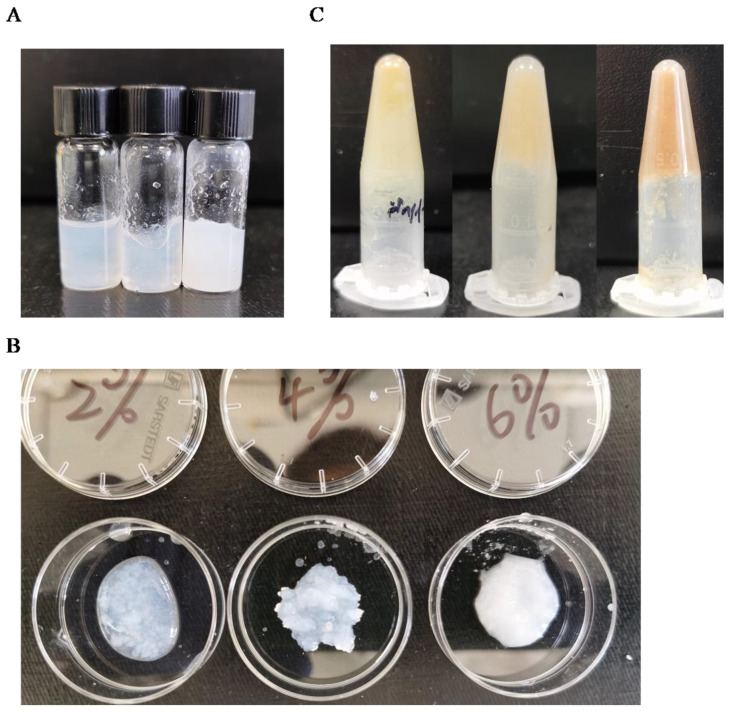
Cross-linking analysis after sonication: (**A**) 2%, 4%, 6% oxidized microcellulose 450 μL with 1 M calcium chloride 50 µL; (**B**) 2%, 4%, 6% oxidized microcellulose detail show; (**C**) 2%, 4%, 6% oxidized wheat straw 450 µL with 1 M calcium chloride 50 µL.

As carboxyl-containing polysaccharides often possess the ability to crosslink with divalent metal ions, various concentrations of oxidized cellulose (2%, 4%, and 6%) were tested for crosslinking with 1 M calcium chloride (Figure 3A). It was observed that lower concentrations of oxidized microcellulose exhibited partial cross-linking (Figure 3B), whereas higher concentrations resulted in sedimentation. Consequently, it was determined that 4% oxidized microcellulose represented the optimal concentration for cross-linking. In contrast, different concentrations of oxidized wheat straw did not exhibit significant variations in cross-linking (Figure 3C), but for comparison purposes, a concentration of 4% was selected.

### 3.4. Rheological Analysis

#### 3.4.1. Viscosity

**Figure 4:**
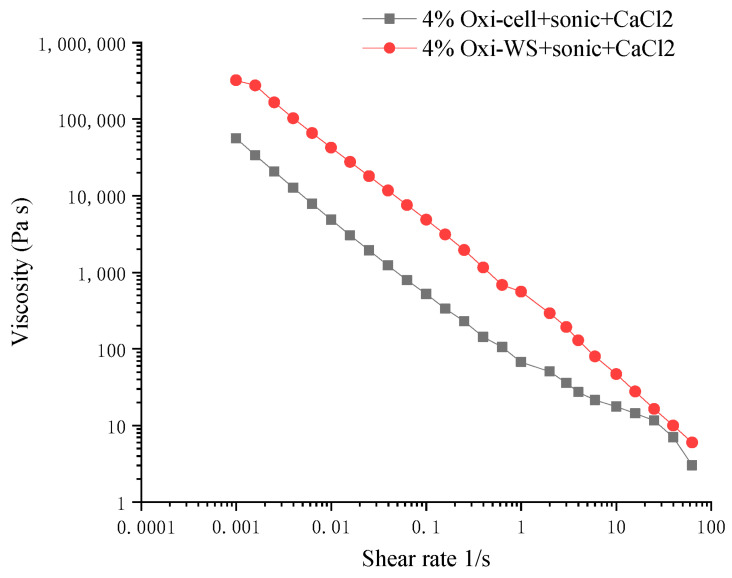
The viscosity analysis after cross-linking. The black line represents 4% oxidized wheat straw, and the red line represents 4% oxidized microcellulose.

Viscosity analysis of 4% oxidized cellulose and oxidized wheat straw revealed a pseudoplastic curve. Subsequent analysis of the solution post crosslinker addition indicated an increase in viscosity. We found that the viscosity of oxidized wheat straw was greater than that of oxidized microcellulose (Figure 4). We also observed a fivefold difference in viscosity for oxidized microcellulose before (851.2 Pa.s) and after (4862 Pa.s) crosslinking at a shear rate of 0.015. The addition of the crosslinker significantly enhances the viscosity of the hydrogel.

#### 3.4.2. Gelation time using G′ and G″

**Figure 5:**
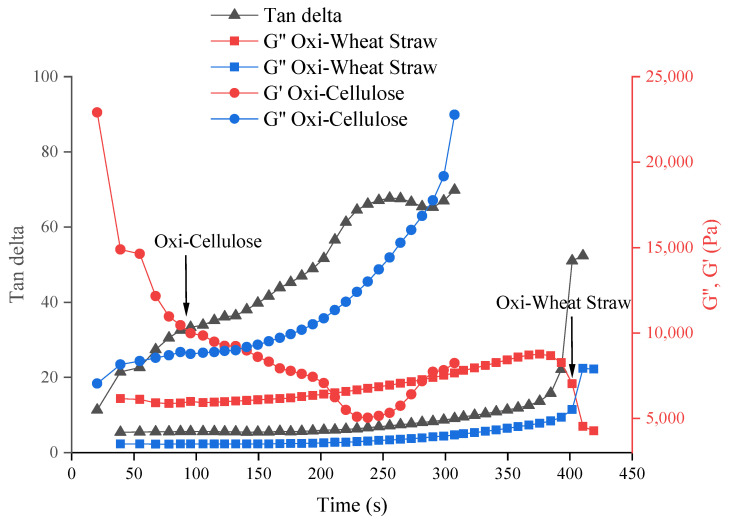
The crosslinking time of different materials with calcium chloride. It is 4% oxidized microcellulose crosslink with 1 M CaCl_2_ (around 2 min) and 4% oxidized wheat straw crosslink with 1 M CaCl_2_ (7 min). The arrows in the graph are the cross-linking points (gelation points).

Following the addition of a crosslinker, the transformation from liquid to hydrogel is evident based on the changes in storage modulus and loss modulus, as illustrated in Figure 5. Both 4% oxidized cellulose and oxidized wheat straw exhibited complete crosslinking within 7 min. Nevertheless, the cross-linking time of oxidized microcellulose is shorter, and the cross-linking process can be completed within 5 min (Figure 5).

### 3.5. Injectability and Printability Analysis

**Figure 6:**
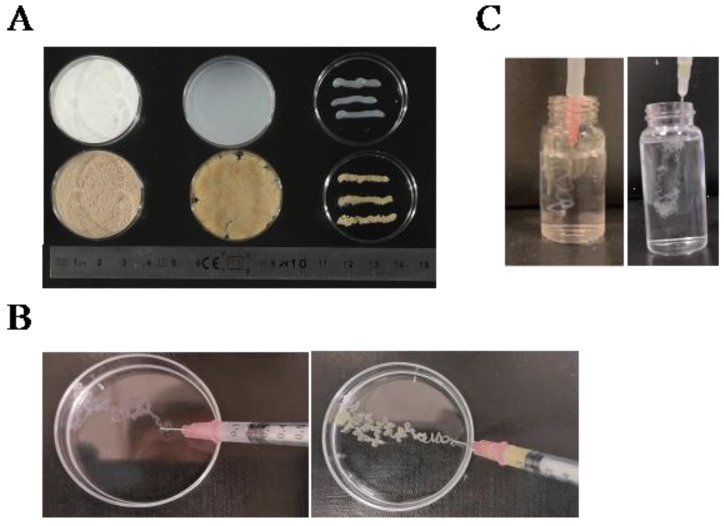
Printability and injectability tests after sonication were conducted as follows: (**A**) 4% oxidized microcellulose (top) and wheat straw (bottom) underwent a printability test under a 19 G (1 mm) diameter needle; (**B**) Printability tests of 4% oxidized microcellulose (left) and wheat straw (right) were performed in a 1 M calcium chloride solution using a needle diameter of 0.4 mm; (**C**) Injectability tests of 4% oxidized microcellulose (left) and wheat straw (right) were carried out in a 1 M calcium chloride solution using a needle diameter of 34 G (0.1 mm).

Initially, we evaluated the extrusibility of 4% oxidized cellulose and wheat straw using a 1 mm diameter needle, finding them to be pliable without a crosslinker (Figure 6A). For printability analysis, the 4% oxidized polysaccharide was extruded into a Petri dish containing 1 M CaCl_2_ using a 27 G (0.4 mm) needle. After five minutes, it was observed that the material had formed a mechanically stable filament (Figure 6B). The printability of the solution (4%) was also assessed using a needle tube and a 27 G (0.4 mm) diameter needle after sonication. The sonicated solution was injected through a 0.4 mm needle into a 1 M CaCl_2_ solution at 24 °C (Figure 6C). After a five-minute wait, shape-stable filaments were obtained at the bottom of the solution, showcasing the potential for injectable hydrogels for potential in vivo applications. To assess injectability, we utilized videos for demonstration purposes. We have added six videos to the supplemental material to show the details. Videos S1 and S2 showcase oxidized and sonicated microcellulose and wheat straw, respectively, being extruded into a Petri dish containing a crosslinker to assess the stability of their morphology. Our results have initially demonstrated its printability, and the next step should be an investigation of 3D bioprinting according to the application. Videos S3 and S4 illustrate the injectable capacity of oxidized cellulose before and after sonication, respectively. Similarly, Videos S5 and S6 demonstrate the injectable capability of oxidized wheat straw before and after sonication. The inverted vial method was employed to showcase changes in viscosity. The viscosity of the vial markedly increased after ultrasonication.

### 3.6. Transmittance Analysis

**Figure 7:**
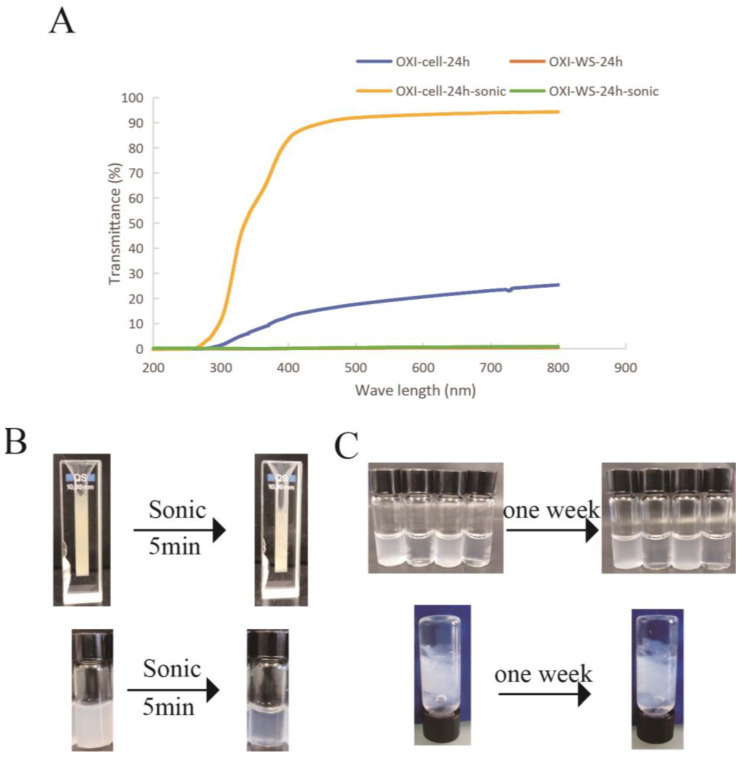
(**A**) Changes in transmittance of 4% oxidized microcellulose and wheat straw before and after sonication. (**B**) Demonstration of oxidized microcellulose and wheat straw transmittance and, (**C**) one-week stability observation of oxidized cellulose solutions after sonication (up) and of oxidized cellulose solutions after sonication and CaCl_2_ crosslink (down).

For bioinks, transparency is a very important feature, and we use transmittance to express transparency. The impact of ultrasound on the transmittance of polysaccharides before and after sonication was examined. After 5 min of sonication, the light transmittance of oxidized microcellulose reached more than 80%, expanding its potential applications in tissue engineering. However, oxidized wheat straw did not show an increase in light transmittance (lower than 5%) following ultrasound treatment (Figure 7). This illustrates that sonication can not only increase the viscosity of the oxidized polysaccharide solution but also increase its light transmittance. Additionally, we observed that the light transmittance of the solution remained unchanged even one week after sonication. This indicates that the nanoparticles generated through ultrasound carry carboxyl groups, ensuring their stability in water solubility. This observation underscores the robust nature of the produced nanoparticles and their sustained stability over time.

### 3.7. Granularity Analysis

**Figure 8:**
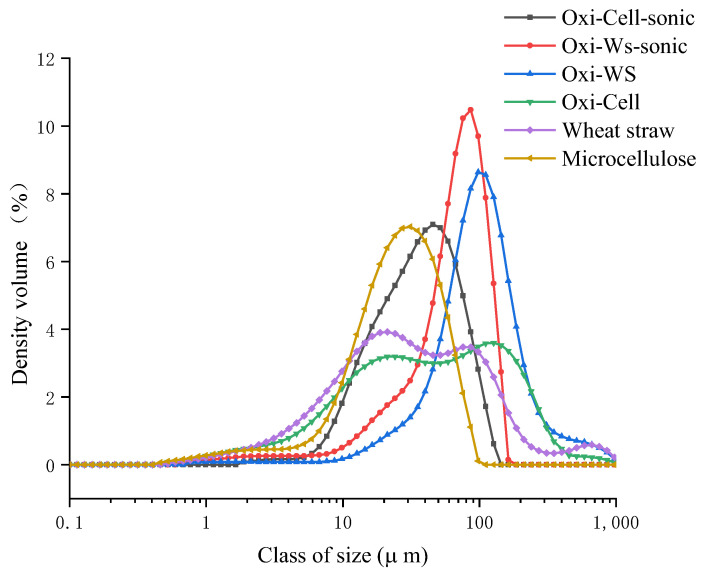
Granulometry analysis (particle sizes) of wheat straw and microcellulose samples before and after oxidation and sonication treatments.

Oxidation and subsequent re-drying lead to the polymerization of nanofibers, causing an increase in particle size that hinders efficient crosslinking with CaCl_2_. Therefore, additional sonication is necessary. The solution underwent drying both before and after oxidation and sonication, followed by a thorough one-hour grinding process. Subsequently, the material was sieved through a 125 micron mesh. The particle size of microcellulose increased from 31.3 µm to 86.2 µm after oxidation, while the particle size of wheat straw increased from 74.6 µm to 138 µm following the oxidation process. Additionally, we noted that sonication led to a reduction in the particle size of the materials. After ultrasonication, the particle size of oxidized cellulose decreased to 44 microns, and for wheat straw, it reduced to 72.4 microns (Figure 8). Our hypothesis suggests that the observed increase in particle size post-oxidation results from re-drying, causing small particles to aggregate and resist dispersion even after an hour of grinding. Therefore, sonication becomes necessary after the oxidation process. The observed decrease in granularity after sonication can be attributed to the disruptive effect of sonication, which breaks down larger particles into numerous nanoparticles. This process of fragmentation leads to a reduction in overall particle size, explaining the observed decrease in granularity after ultrasonication.

## 4. Discussion

Wheat straw biomass represents an underutilized raw material that has the potential to make a substantial contribution to sustainable development. The development of a new generation of hydrogels based on modified cellulose holds great significance for resource regeneration and cost reduction. In this study, wheat straw was utilized as a raw material, and carboxyl groups were introduced through TEMPO oxidation to produce a novel hydrogel. The properties of this hydrogel were then compared with those of commercial cellulose sourced from cotton.

In the comparison of FTIR results, it was observed that with the increase in oxidation time, the corresponding carboxylic acid peak at 1720 cm^−1^ gradually transformed into carboxylate groups, manifesting as a peak at 1610 cm^−1^ [34]. The presence of peaks at 1454 cm^−1^ (C–H and CH_2_ bending vibration in lignin) and 1245 cm^−1^ (C–O stretching vibration of acetyl in hemicellulose) was simultaneously observed in the peak plot analysis of wheat straw [35]. Due to the absence of a chromophore in carboxylic acid, UV absorbance detection is rarely utilized. Ion exchange chromatography detection, offering high sensitivity, is more commonly employed [36]. In the 3D printing process, bioinks have distinct requirements before, during, and after extrusion. Prior to extrusion, key characteristics include viscosity and biocompatibility. During the extrusion stage, the focus is on minimizing shear stress [37]. Evaluation at this stage includes assessing the physiological stability and cell viability of the 3D-printed structure. Post-extrusion evaluation primarily involves examining mechanical support capacity and degradability. The viscosity test results indicated a typical non-Newtonian fluid behavior with fluidity increasing at high shear rates. In 3D printing, the structures produced can be immersed in a bath containing a crosslinker or exposed to physical crosslinking conditions, such as freeze–thaw cycles. This study indicates that 4% oxidized wheat straw exhibits the potential for printing in a solution containing a crosslinker such as CaCl_2_ [38]. The performance of bioinks was characterized by measuring the storage modulus and loss modulus of bioinks before and after cross-linking. It is desirable for the gel to form rapidly after extrusion, ensuring the stability of its shape and structure [39]. Viscosity serves as a crucial rheological parameter in assessing the printability of bioinks. Lower viscosity can compromise shape fidelity, while higher viscosity can hinder extrusionability. In extrusion-based bioprinting, the stable formation of bioink filaments is vital for printing multilayer structures [40]. In this experiment, 4% oxidized polysaccharides were successfully cross-linked in less than 100 seconds, showcasing the feasibility of 3D printing applications. After only five minutes of sonication, the light transmittance of the solution was significantly improved, because the ultrasound reduced the chain length of the polysaccharide, resulting in a greatly reduced scattering of light, thus improving the light transmittance [41]. Biodegradable polymers are suitable for developing injectable implants because of their biocompatibility, sterility, and controlled release properties [42]. The characterization of light transmittance serves as a reflection of the material’s potential applications. After 5 min of ultrasonication, the light transmittance of the material exhibited significant improvement, expanding the potential application range of the hydrogel for the tissue engineering field [43].

## 5. Conclusions

In this paper, two new hydrogels were successfully modified through TEMPO oxidation, showcasing their future applicability in 3D printing. Various physical and chemical characterizations, including oxidation degree, gel ability, injectable ability, printable ability, viscosity, light transmittance, etc., were conducted. This study affirms the potential for distinct value chains of cellulose, hemicellulose, and lignin, as well as the recycling of lignocellulosic biomass by-products. It serves as a valuable reference for the development of green, safe, and cost-effective hydrogels. Next, we will conduct tests on two new hydrogels at different concentrations to explore their potential applications in various fields and acquire a comprehensive understanding of both materials.

## Figures and Tables

**Table 1:** Oxidized microcellulose and wheat straw oxidation degree.

Sample Name	σ	Degree of Oxidation
Oxi-cell 3 h	0.000675	56%
Oxi-cell 24 h	0.000825	69%
Oxi-wheat straw 3 h	0.000675	56%
Oxi-wheat straw 24 h	0.00075	63%

## Data Availability

Data are contained within the article and Supplementary Materials.

## Supplementary Materials

The following are available online at https://www.mdpi.com/article/10.3390/polym16040530/s1, Video S1: Printability Oxi-cell-24 h-after sonic; Video S2: Printability Oxi-Wheat straw-24 h-after sonic; Video S3: injectability Oxi-cell-24 h; Video S4: injectability Oxi-cell-24 h-after sonic; Video S5: injectability Oxi-Wheat straw-24 h; Video S6: injectability OXI-Wheat straw-24 h-after sonic.
